# The impact of familial risk and early life adversity on emotion and reward processing networks in youth at-risk for bipolar disorder

**DOI:** 10.1371/journal.pone.0226135

**Published:** 2019-12-12

**Authors:** Lindsay C. Hanford, Kristen Eckstrand, Anna Manelis, Danella M. Hafeman, John Merranko, Cecile D. Ladouceur, Simona Graur, Alicia McCaffrey, Kelly Monk, Lisa K. Bonar, Mary Beth Hickey, Tina R. Goldstein, Benjamin I. Goldstein, David Axelson, Genna Bebko, Michele A. Bertocci, Mary Kay Gill, Boris Birmaher, Mary L. Phillips

**Affiliations:** 1 Western Psychiatric Institute and Clinic, University of Pittsburgh Medical Center, University of Pittsburgh, Pittsburgh, Pennsylvania, United States of America; 2 Psychiatry, Sunnybrook Health Sciences Centre, Toronto, Canada; 3 Pharmacology and Toxicology, University of Toronto, Toronto, Canada; 4 Nationwide Children’s Hospital and The Ohio State College of Medicine, Columbus, Ohio, United States of America; Assistance Publique Hopitaux de Marseille, FRANCE

## Abstract

A recently developed risk calculator for bipolar disorder (BD) accounts for clinical and parental psychopathology. Yet, it is understood that both familial predisposition and early life adversity contribute to the development of BD. How the interplay between these two factors influence emotion and reward processing networks in youth at risk for BD remains unclear. In this exploratory analysis, offspring of BD parents performed emotion and reward processing tasks while undergoing a fMRI scan. Risk calculator score was used to assess risk for developing BD in the next 5 years. Environmental risk was tabulated using the Stressful Life Events Schedule (SLES). Emotion and reward processing networks were investigated for genetic and/or environment interactions. Interaction effects were found between risk calculator scores, negative SLES score and activity in right amygdala and bilateral fusiform gyri during the emotion processing task, as well as activity in the fronto-, striatal, and parietal regions during the reward processing task. Our findings are preliminary; however, they support the unique and interactive contributions of both familial and environmental risk factors on emotion and reward processing within OBP. They also identify potential neural targets to guide development of interventions for youth at greatest risk for psychiatric disorders.

## Introduction

Neurodevelopmental models for Bipolar Disorder (BD) posit the involvement of both genetic predisposition, such as having a parent diagnosed with BD, and life stressors, such as early life adversity [1]. The interplay between these two factors results in a variety of epigenetic changes that impact different neurodevelopmental processes. These include altered hypothalamic-pituitary-adrenal axis activity [2, 3], altered immunological response [4, 5], and altered development of emotion and reward processing circuitry [6–8]. Repeated exposure to stress may worsen emotional and behavioral problems in youth, and predispose to development of psychiatric disorders such as BD [9–11]. Mood symptoms in BD are thought to be a function of dysregulation within emotion and reward processing networks [7, 8, 12, 13]. A better understanding of the unique contributions and interplay between genetic and environmental risk factors on the development of emotion and reward processing networks may thus help to identify specific neural mechanisms associated with each of these influences, and, in turn, yield neural targets to guide preventative strategies and targeted interventions for youth at the greatest risk of future psychiatric disorders.

BD has one of the highest heritability rates among psychiatric disorders [14–17]. Offspring of parents diagnosed with BD (OBP) have a tenfold increased risk for developing BD across their lifetime relative to individuals without a family history of BD [17–19]. Moreover, those who develop psychopathology early on are more likely to develop BD in the future [20–23]. A recently developed predictive risk calculator supports this idea by showing good discrimination (AUC 0.76) for the 5-year outcome of those OBP who would go on to develop BD [20]. Here, the greatest predictor of future BD in OBP was parental age at onset of mood symptoms [20], highlighting that both genetic risk and early onset of BD in the parent are important risk factors contributing to the development of BD in offspring.

Dysregulation within emotion and reward processing networks are thought to underlie mood symptoms in BD [7, 8, 12, 13]. As such, these networks would be among the best avenues for exploring the underlying architecture for the development of BD. In the emotion processing network, the amygdala plays a central role for the detection of emotional cues [24–26]. The medial prefrontal cortex (mPFC) and insula are important for subjective salience and value assignment, the dorsolateral PFC (dlPFC) for cognitive control, and ventrolateral prefrontal cortex (vlPFC) and anterior cingulate cortex (ACC) for emotion regulation and conflict monitoring [7, 8, 12, 13]. Together, these prefrontal regions provide feedback as a top-down modulatory mechanism over amygdala responsivity [7, 8, 12, 13]. This top-down modulation is dysregulated in both youth with and at-risk for BD [27–32]. Specifically, amygdala hyperactivity [27–30] and greater positive functional connectivity between amygdala and orbital and ventral prefrontal cortices [27, 31] were observed in OBP relative to healthy control youth across a wide variety of emotion processing tasks. In studies where a comparative group with BD was included, OBP exhibited more similar patterns of neural response to youth with BD than to healthy offspring [27, 28, 30]. In other studies, OBP have shown lower prefrontal cortical activity [33, 34], lower amygdala-vlPFC functional connectivity [29, 32], and lower amygdala-ACC functional connectivity [29] during emotion processing relative to healthy offspring. Variability in these results may be due to heterogeneity of OBP populations.

In the reward processing network, the ventral striatum (VS) supports reward valuation and response to motivational cues [35–41]. Prefrontal regions including the mPFC and insula process subjective experience of reward and motivation, while the vlPFC subserves learning and decision-making components of reward processing [12, 36, 42]. Greater VS activity and reduced modulatory connectivity of the VS have been observed in individuals with BD [43–46]. Greater orbitofrontal cortical [47, 48], amygdala [48] and frontal pole activity [49] and greater negative VS-vlPFC functional connectivity during reward processing [49] have been shown in OBP relative to a healthy control offspring group. OBP also showed differential patterns of pregenual ACC-vlPFC connectivity: reduced during reward anticipation, and increased during loss anticipation, relative to healthy control youth [47]. Taken together, these studies indicate aberrant activity and connectivity in emotion and reward processing neural circuits in OBP, which may represent neural markers of risk for future BD. Inconsistencies in some of these findings may result from the inclusion of heterogeneous OBP populations, of particular interest, the level of exposure to early life adversities, or stressful life events (SLEs). To date, however, the impact of this kind of adversity on emotion and reward circuitry functioning in OBP remains to be determined.

Living with a person diagnosed with any psychiatric disorder increases the likelihood for experiencing SLEs including: social/emotional burden, financial/legal burdens, exposure to violence, and lower quality of parental care [50, 51]. Amygdala hyperactivity, during emotion processing and stress-related tasks, has been reported within a variety of healthy and psychiatric populations, and across a range of SLE types [52–60]. In the absence of stable caregiving, required for typical emotional development [60], offspring show immature, more positive amygdala-PFC functional connectivity [61]. Similarly, individuals exposed to early SLEs showed reduced striatal activity during reward processing [62, 63]. Moreover, activity in orbitofrontal cortex (OFC), insula, ACC, and amygdala may mediate the relationship between SLEs and anxiety in an otherwise healthy adolescent population during emotion processing [54]. As such, emotion and reward processing networks appear to be vulnerable to early adversities [64, 65], and may mediate risk for future psychopathology [66].

Thus, more broadly, both genetic and environmental factors alter activity and connectivity within emotion and reward processing networks. Yet, the interplay and specific contributions of these factors on these networks in OBP remains unclear. In this exploratory study, we examined OBP with varying risk for future BD, and a range of exposure to negative SLEs to examine the interaction and separate effects on (1) emotion and (2) reward processing networks to better elucidate how these factors might exacerbate aberrant network functioning. While previous work has established emotion and reward processing deficits, and associated aberrant network functioning in OBP, to our knowledge, no studies have examined the specific contributions of SLEs, or the interplay of genetic and environmental risk factors for BD in OBP. We hypothesized that relative to healthy youth offspring of healthy parents, the greatest magnitude of abnormal functioning of emotion and reward processing circuits, in particular in amygdala, VS and prefrontal cortical regions would be observed in OBP with highest predictive risk, as assessed by the risk calculator, and with the greatest exposure to negative SLEs.

## Methods

This study was conducted in accordance with the Human Research Protection Office at the University of Pittsburgh. Written informed consent and assent were obtained from parent and child, respectively. All participants received monetary compensation for their time and expenses. Study details including clinical assessments, and functional tasks have been described previously within this sample [29, 49].

### Participants

Offspring of bipolar parents (OBP) were recruited as part of the larger Bipolar Offspring Study; an on-going longitudinal study at the University of Pittsburgh examining biological markers related to risk and outcome in these offspring (NIMH060952) [67]. Similarly, a subset of healthy control offspring (HCO) from the Longitudinal Assessment of Manic Symptoms (LAMS) study; a longitudinal study investigating the outcome and variability of behaviors related to emotion dysregulation were included in this study (NIMH073953) [68]. All participants were (1) between the ages of 7–17 years, (2) proficient in the English language, (3) free of any severe medical illnesses, (4) neurological conditions, (5) mental health concerns including substance or alcohol use disorders, or (6) pervasive developmental disorders. Participants were excluded if they had an IQ<70, poor acuity (<20/40), or had any MRI contraindications.

The current sample included subjects from previous emotion processing [29] and reward processing [49] analyses (22 OBP and 22 HC), as well as 3 new OBP subjects. This totals twenty-five OBP [14.1± 2.4 years, 40% female, 100.5± 15.0 IQ] and 22 HCO [13.7± 1.8 years, 50% female, 104.8± 13.3 IQ] with high quality functional imaging data were included in the final sample. There were no significant differences in age, sex or IQ between groups.

### Clinical assessments

Parent and child were interviewed using the Kiddie Schedule for Affective Disorders -Present and Lifetime (K-SADS-PL)[69] to confirm the presence of any diagnoses in the child. Offspring with a diagnosis of BD, autism, schizophrenia, or substance abuse were excluded. Interrater reliability for diagnoses ascertained through the KSADS-PL was >0.8. Additionally, all cases were reviewed by a child psychiatrist (B.B.). Parents were assessed using the Structured Clinical Interview for the DSM-IV (SCID)[70] to confirm a diagnosis of BD (any type) in the OBP group, and a detailed clinical assessment was used to confirm no past or current diagnosis in the parents of the HCO group.

Well-validated symptom scales, including the KSADS-PL Depression Rating Scale (KDRS)[69, 71], the KSADS-PL Mania Rating Scale (KMRS)[69, 71], the Children’s Affect Liability Scale (CALS)[72], and Screening for Child Anxiety Related Disorders (SCARED) scale [73, 74], and the Children’s Global Assessment Scale (CGAS)[75], were administered to both child and to the parent about their child during a diagnostic interview. Additional information on clinical assessments and covariate information can be found in *Supplementary Methods*.

### Risk calculator score

Recently, our group developed a predictive risk calculator to assess the probability for OBP to develop BD within the next 5 years (http://www.pediatricbipolar.pitt.edu) [20]. This tool showed very good discrimination for OBP who went on to develop BD (AUC of 0.76, 95%CI:0.71–0.82). This performance is comparable to other risk calculators used in medicine [76–78]. Scores were calculated using a modified KMRS score, modified KDRS score, child reported SCARED score, child reported CALS score, CGAS score, offspring age and parental age at mood disorder onset as an earlier onset in the parent is likely to have familial transmission [20, 79]. It is worth noting that parental age at mood disorder onset was one of the strongest contributing variables for calculating risk score, emphasizing the importance of familial risk for BD [20].

In our sample, predictive risk calculator scores ranged from 0.008–0.24 with a mean and standard deviation of 0.071 ± 0.066. Risk Calculator values range may range from 0 to 1, and represent a percentage likelihood of converting over the next 5 years [20]. Since conversion is low on average, however, still represent a greater risk than the general population risk (1–2%)[17–19]. Currently, this calculator is available for use only within the OBP population, and therefore has limited ability for comparison among other at-risk or healthy populations, however, we would expect healthy controls to show very low risk of converting and would therefore hold values close to 0. For this study, this tool was used to identify those OBP at the greatest risk (higher risk calculator score) of developing BD in order better establish biological markers most related to risk.

### Negative stressful life events schedule (nSLES) score

Exposure to negative SLEs in the past year were tabulated using the adult and child, or adult and adolescent versions of the Stressful Life Events Schedule (SLES)[80]. This questionnaire has been validated and accounts for both presence (e.g. “In the past 12 months, I was bullied at school or in my neighborhood”) and impact (e.g. “How did this affect you? 1-Not at all, 2-A little, 3-Somewhat, 4-A lot”) of events. Events which scored >2 on impact were tallied and used to indicate negative SLEs [80, 81]. Participants who had experienced sexual abuse (n = 2), or had a >12-month gap between scan and self-report SLES acquisition (n = 3) were excluded. Negative SLES (nSLES) scores were acquired for OBP, and were used as an indication of negative environmental event exposure in the last year. In our OBP sample, nSLES scores ranged from 0–8 with a mean and standard deviation of 2.7±2.3.

### Functional tasks

Emotion and reward processing tasks have been described previously [29, 49], and are described in the *Supplementary Material*. Briefly, during the emotion processing task, participants observed dynamically changing emotional faces while being asked to identify a color flash that appeared within each stimulus, thereby eliciting implicit emotion processing. The main conditions included: happy, sad, angry, fearful and shape (control) conditions. The main contrast of **all emotions versus shapes** was used for this task.

During the reward processing task, participants were asked to guess whether the next number presented would be above or below 5. Participant had a chance to gain money (reward condition) or lose money (loss condition) based on the accuracy of their answer. As a control condition, where participants were asked to push the button when an asterisk appeared on the screen. The main contrast of **reward versus control** condition was used for this task, as this has been shown to be associated with greater engagement of reward circuitry rather than loss in at-risk youth [82].

### Data analysis

Images were analyzed using FMRIB’s Software Library (FSL:v5.0 www.fmrib.ox.ac.uk/fsl)). Full details on the preprocessing and analysis of activity and functional connectivity have been previously described elsewhere [29, 49], and appear in the *Supplementary Materials*.

First-level (subject-level) general linear models included functional task regressors to examine whole-brain stimulus-related activity, and to extract the time series of seed regions in functional connectivity analyses. Seed regions were defined by the Harvard-Oxford Structural Atlas. Voxels with at least 99% probability were selected to be part of the mask, and were subsequently binarized. For the emotion processing task, bilateral amygdala were chosen as the seed region based on its role within the network, as well as its robust task activation [26, 29]. Likewise, bilateral ventral striatum was chosen as the seed region for the reward processing task [40, 49, 83].

Functional connectivity analyses were conducted using psychophysiological interaction (PPI) methods, to compare correlations of brain activity to a given seed region across different psychological or task conditions [84, 85]. PPI first-level models included: the respective psychological stimulus contrast as described above for each task, one physiological regressor (the mean time course extracted from the seed region), and the respective interaction terms between psychological and physiological regressors.

All within-group general linear models were conducted using FLAME1 (FMRIB’s local analysis of mixed effects), where the effects of sex, age and IQ were regressed out. The main contrasts of all emotions versus shape, and reward versus control conditions were used for emotion and reward tasks, respectively. Mean-centered risk calculator score, and mean-centered negative SLES (nSLES) score and risk calculator-by-nSLES score interaction terms were included in the models as covariates of interest.

To identify brain regions that were sensitive to the interaction effect of risk calculator and nSLES scores, we used analysis of covariance (ANCOVA) models on measures of brain activity and functional connectivity within OBP. Similarly, ANCOVA models were used to uncover the main effects of risk calculator score or nSLES score (e.g. the relationships between risk calculator score and brain metrics were estimated while accounting for the effects of nSLES score and interaction effects between these two variables).

### Statistical analyses

Graphical depictions of these interactions were plotted using the visreg package [86] within R version 3.3.1 software (http://www.r-project.com). To do this, mean blood oxygen level dependent (BOLD) signal and functional connectivity values were extracted for each significant region showing an interaction effect between risk calculator score and nSLES score. This was done for both the emotion and reward tasks within the OBP group. Once defined within the OBP group, BOLD signal and functional connectivity metrics were extracted in the same regions within the HCO group. As risk calculator and nSLES scores were not available for HCO, neuroimaging measures in OBP were HCO mean-adjusted for all analyses. In this way, we were able to interpret mean BOLD activity and connectivity of OBP that was greater or less than that expected in HCO. Additionally, given that OBP and HCO comparisons have already been made in this sample [29, 49], this comparison was not a priority for this paper.

Multiple comparisons were corrected for using Gaussian Random Field theory (GRF): Z-statistic threshold at z>2.3 (uncorrected voxel-wise p<0.01) and a family-wise error-corrected cluster significance threshold of alpha = 0.01/6 comparisons (3- risk models: interaction model, risk calculator model, environmental risk model; by-2 imaging methods: activity, functional connectivity) = 0.0017 [87]. Beyond interaction model statistics, we did not have sufficient power to further separate groups based on high/low risk calculator score, or high/low nSLES score, as such our interpretations of the interaction effects are purely descriptive.

## Results

### Demographics and clinical information

The majority of parents with bipolar disorder had BD type I (n = 17), others had BD type II (n = 8). In addition to BD, most of these parents had comorbid disorders; most commonly, a specific phobia (n = 16), panic disorder (n = 14), or a substance use or abuse (n = 15). A total of 8 OBP had a diagnosis at the time of the scan (ADHD n = 4, MDD n = 2, mood disorder NOS n = 1, GAD n = 1, Eating Disorder n = 2, ODD n = 1, Tourette’s Syndrome n = 1). Full details on current and lifetime diagnoses, as well as age of onset for both parent and child can be found in Table 1.

**Table 1:** Demographic and clinical information.

	**Present (Age of Onset Range)**	**Lifetime (Age of Onset Range)**
***Offspring diagnoses***		
**Total number with at least one diagnosis**	**8**	**16**
Major depressive disorder	2 (14–16 yrs)	1 (8 yrs)
Mood disorder, not otherwise specified	1 (12 yrs)	4 (6–12 yrs)
Attention deficit hyperactivity disorder	4 (5 yrs)	4 (3–6 yrs)
Anxiety disorder (general or separation)	1 (6 yrs)	8 (2–14yrs)
Specific phobias	1 (15 yrs)	2 (8m–4yrs)
Eating disorder	2 (5–14 yrs)	0
Oppositional defiant disorder	1 (5 yrs)	4 (4–5 yrs)
Adjustment disorder	1 (14 yrs)	2 (7–11 yrs)
Phonological disorder	1 (7 yrs)	0
Tourette’s or Tic disorder	1 (11 yrs)	2 (9–10 yrs)
Enuresis	1 (5 yrs)	4 (2–5 yrs)
***Parental diagnoses***	**Present and Lifetime (Age of Onset Range)**	
Bipolar disorder (Type I)	17 (11–30 yrs)	
Bipolar disorder (Type II)	8 (9–35 yrs)	
Attention deficit hyperactivity disorder	11 (5–11 yrs)	
General anxiety disorder	15 (4–44yrs)	
Phobias	17 (3–39 yrs)	
Panic disorder	12 (13–36 yrs)	
Eating disorders	5 (14–44 yrs)	
Substance use/abuse disorders *	17 (13–39 yrs)	
Post-traumatic stress disorder	13 (13–37 yrs)	
Obsessive compulsive disorder	9 (13–36 yrs)	
Personality disorders (borderline)	13 (16–18 yrs)	
Oppositional defiant disorder	11 (4–16 yrs)	

M = months, NA = Not available, Yrs = Years

* substances included cannabis (n = 6), alcohol (n = 12), cocaine (n = 4), or opiod (n = 3), un-specific polysubstance (= 2).

Predictive risk scores did not differ significantly between those with or without a diagnosis (p = 0.08), but, as expected, were moderately positively correlated with nSLES scores (Spearman’s rho = 0.53, p<0.01). Risk calculator score or nSLES score did not differ between OBP of parents diagnosed with BD type I or type II. We also found significantly higher risk calculator scores in females compared to males (Mann Whitney U = 24, n_1_ = 15, n_2_ = 10, p<0.01). No other significant relationships were found between age, sex or IQ and predictive risk scores or nSLES scores.

#### Risk calculator score and nSLES score on whole-brain activity during emotion processing: Interactions and main effects

**Fig 1:**
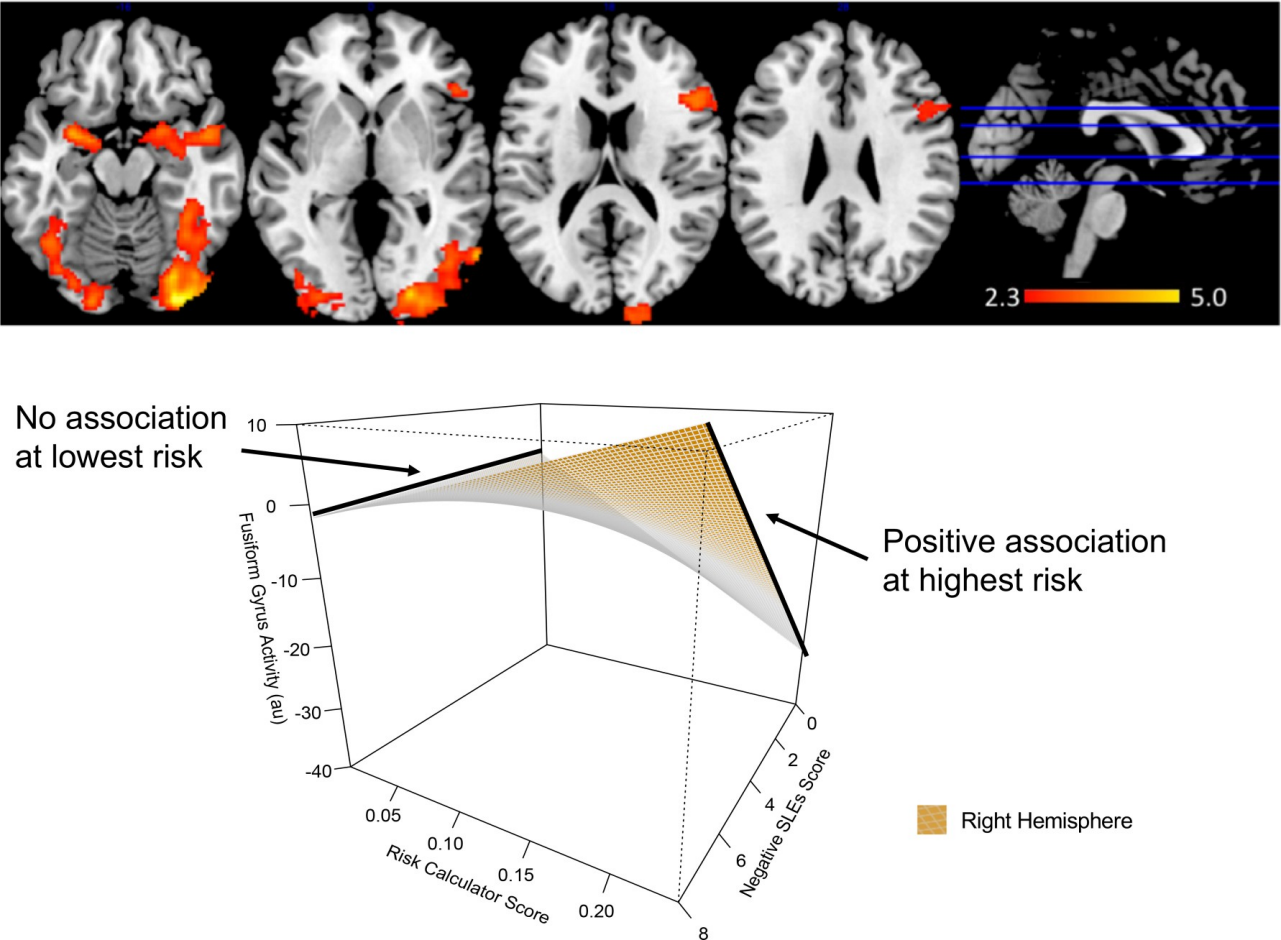
Interaction effects of risk calculator score and negative stressful life events schedule (nSLES) score on whole-brain activity during emotion processing (top). Positive interactions between risk calculator score, nSLES score and activity were found within 3 clusters after correction for multiple comparisons. A graphical representation of this interaction in the right fusiform gyri is presented here (bottom). Higher risk calculator score showed a greater positive association between activity and nSLES score. A full set of these interaction plots can be found in the supplementary. * au = arbitrary units All results were corrected for using Z-statistic threshold at z>2.3, pFWE<0.0017. A contrast of all emotions versus shape conditions was used. Activity values were mean adjusted using a healthy control sample.

Higher risk calculator score showed greater positive associations between number of recent exposure to negative SLEs and activity within bilateral fusiform gyri [R: Z = 5.0, p<0.001; L: Z = 3.6; p<0.001], and the right amygdala [R: Z = 4.1; p<0.001] to all emotions versus shapes conditions (see Fig 1, Table 2, section I). A full set of interaction plots can be found in the supplementary material. There was no association between nSLES score and activity in these regions at lower risk calculator score. Risk calculator score alone showed positive relationships with activity in bilateral lateral occipital cortices [R: Z = 3.7; p<0.001; L: Z = 4.0 p = 0.001], and a negative relationship in the right occipital pole [Z = 4.0; p = 0.002](Table 2: section I). There were no significant associations with nSLES score alone.

**Table 2:** The interaction and main effects of genetic (risk calculator score) and environmental (negative stressful life events score) factors on activity and functional connectivity of emotion processing task.

**Region**	**x,y,z**	**z**	**p**	**size (# of voxels)**
**I. Activity during Emotion Processing task**				
*Risk calculator score*				
Right lateral occipital cortex	16, -76, 48	3.7	2.4e-5	1204
Left lateral occipital cortex	-28, -60, 64	4.0	0.00010	1037
Right occipital pole	24, -90, 2	4.0	0.00016	987
*Negative stressful life events (nSLE) score*				
No significant results				
*Risk score by nSLE score*				
Right temporal occipital fusiform cortex	30, -92, -16	5.0	3.2e-14	4337
Right amygdala, superior temporal gyrus	32, -4, -30	4.1	2.2e-6	1500
Left temporal occipital fusiform cortex	-30, -90, -6	3.6	3.5e-6	1442
**II. Functional Connectivity during Emotion Processing task**				
**Region**	**x,y,z**	**z**	**p**	**size (# of voxels)**
*Risk calculator score*				
Bilateral medial orbitofrontal cortex	6, 38, -18	4.0	0.00011	802
Right lateral occipital cortex	42, -88, 16	4.0	2.4e-7	1388
*Negative stressful life events (nSLE) score*				
Bilateral superior parietal lobule/ lateral superior occipital cortex	10, -40, 80	3.3	0.00046	683
*Risk score by nSLE score*				
Right lateral occipital cortex, occipital pole	40, -72, 4	4.0	2.8e-8	1622
Left lateral occipital cortex, occipital pole	-30, -94, -6	3.6	2.9e-6	1140

Size is measured as number of voxels (2x2x2mm).

#### Risk calculator score and nSLES score on whole-brain functional connectivity during emotion processing: Interactions and main effects

**Fig 2:**
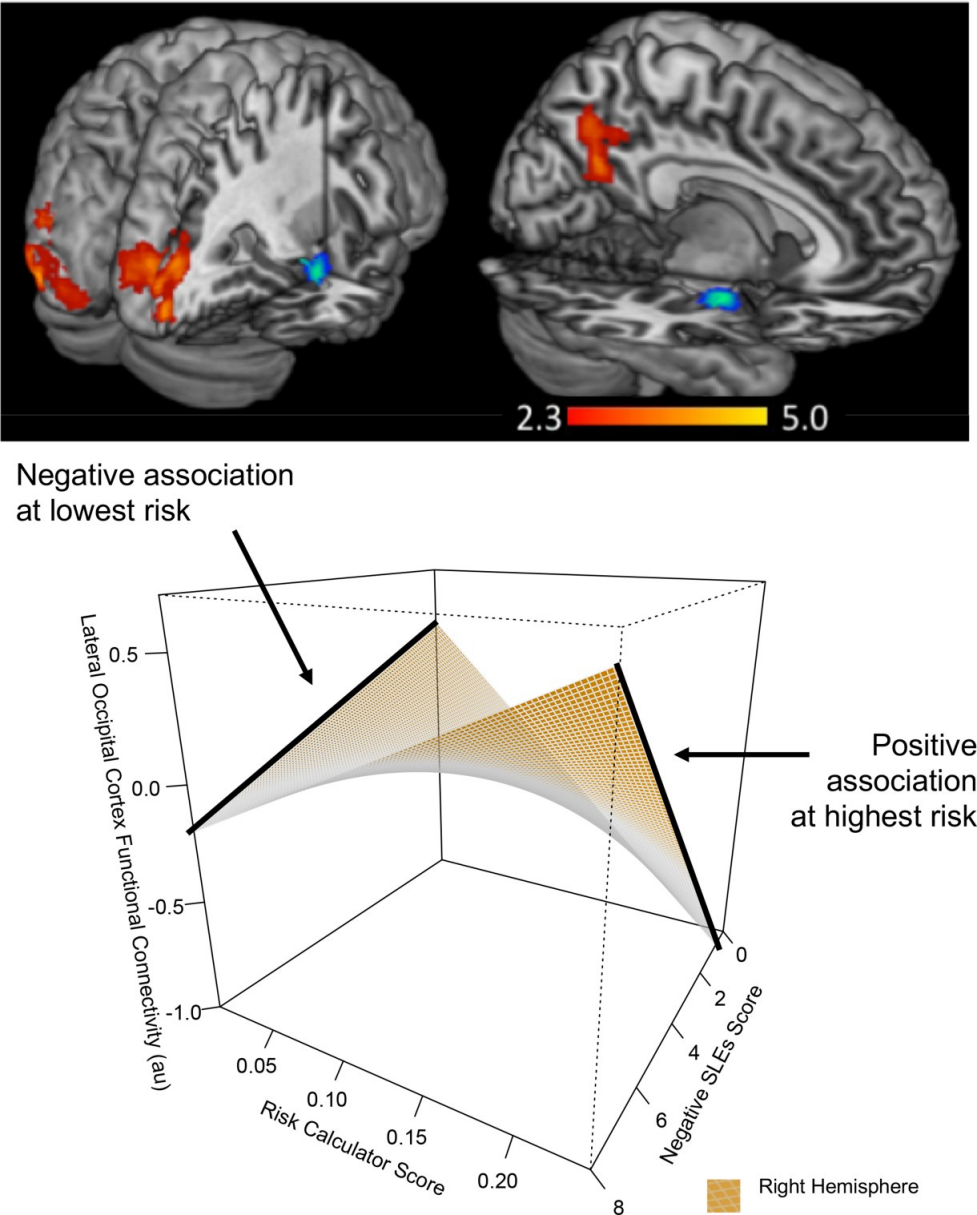
Interaction effects of risk calculator score and negative stressful life events schedule (nSLES) score on whole-brain functional connectivity to bilateral amygdala during emotion processing (top). Positive interactions between risk calculator score, nSLES score and activity were found within 2 clusters after correction for multiple comparisons. A graphical representation of this interaction in the right lateral occipital cortex is presented here (bottom). Higher risk calculator score showed a greater positive association between functional connectivity and nSLES score, which was not present at low risk calculator score. A full set of these interaction plots can be found in the supplementary. * au = arbitrary units All results were corrected for using Z-statistic threshold at z>2.3, pFWE<0.0017. A contrast of all emotions versus shape conditions was used. Functional connectivity values were mean adjusted using a healthy control sample.

Lower risk calculator scores showed a weaker negative association between nSLES score and functional connectivity between bilateral amygdala (seed region) to the right [Z = 4.0; p<0.001] and left [Z = 3.6; p<0.001] lateral occipital cortex to all emotions versus shapes (see Fig 2, Table 2, section II). This association (between nSLES score and function connectivity in these regions) was positive at higher risk calculator scores. Risk calculator score showed a positive relationship with functional connectivity between bilateral amygdala and bilateral medial orbitofrontal cortex [Z = 4.0; p = 0.001], and a negative relationship with functional connectivity of bilateral amygdala and right lateral occipital cortex [Z = 4.0; p<0.001](Table 2: section II). NSLES score showed a positive relationship with functional connectivity between bilateral amygdala and a cluster spanning regions of bilateral superior parietal and lateral superior occipital cortices [Z = 3.3; p = 0.005] (Table 2: section II).

#### Risk calculator score and nSLES score on whole-brain activity during reward processing: Interactions and main effects

**Fig 3:**
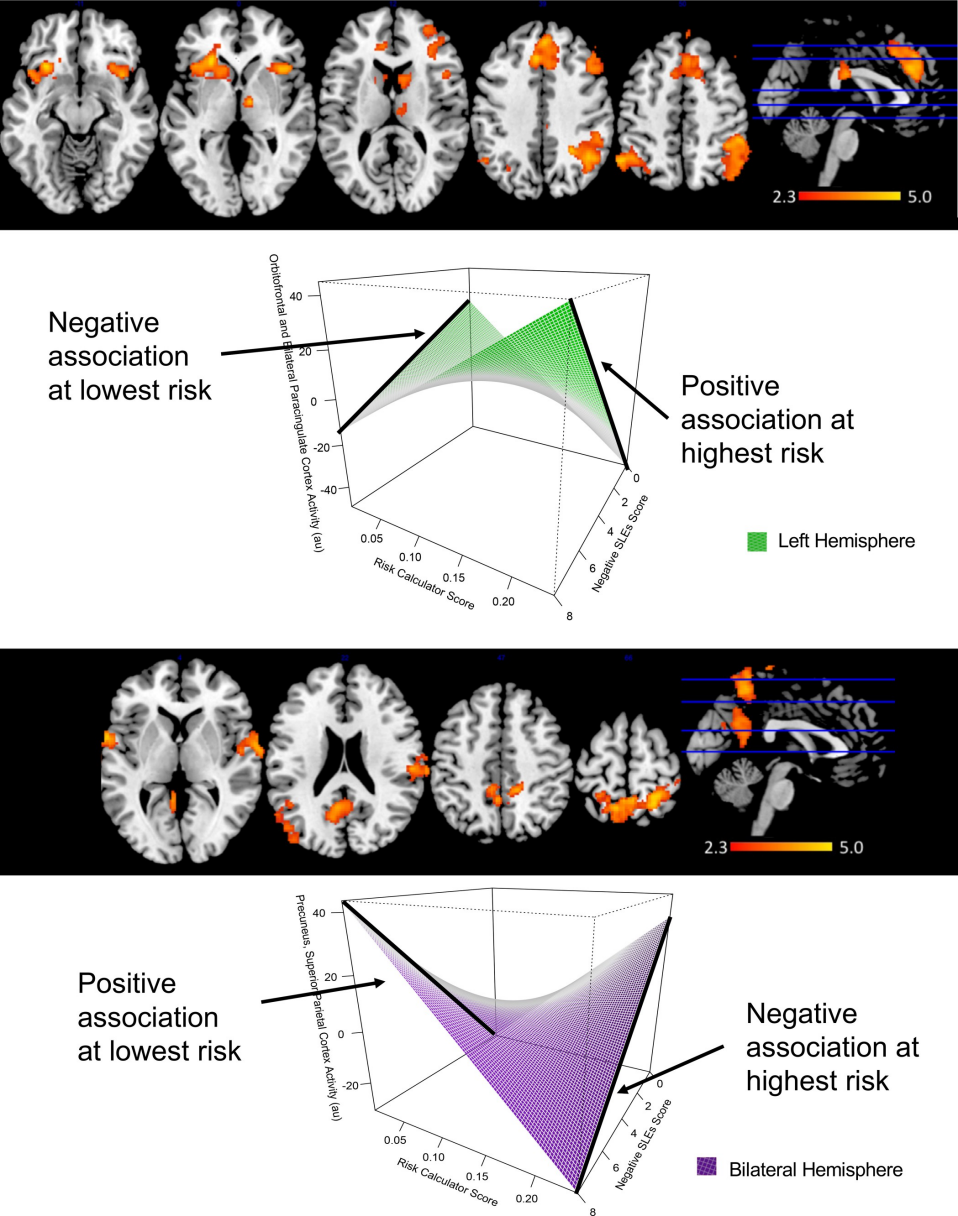
Interaction effects of risk calculator score and negative stressful life events schedule (SLES) score on whole-brain activity measures during reward processing. A) Positive interactions between risk calculator score, nSLES score and activity were found within 5 clusters. A graphical representation of these interactions has been displayed for one large cluster spanning the left orbitofrontal cortex, bilateral paracingulate, left caudate, putamen, and insular cortex. At higher risk calculator score greater positive associations between activity and nSLES score were found, while this relationship was inversed at low risk calculator score. B) Negative interactions between risk calculator score, nSLES score and activity between bilateral amygdala were found within 4 clusters. A graphical representation of this interaction is displayed one cluster spanning bilateral precuneus and superior parietal cortex. In this case, at higher risk calculator score a negative association between activity and nSLES score, while this relationship was positive at low risk calculator score. A full set of these interaction plots can be found in the supplementary. * au = arbitrary units All results were corrected for using Z-statistic threshold at z>2.3, pFWE<0.0017. A contrast of reward vs control conditions was used in all cases. Activity values were mean adjusted using a healthy control sample.

With higher risk calculator scores, the association between higher nSLES score and activity of bilateral supramarginal and angular gyri [R: Z = 4.4, p<0.001; L: Z = 4.0; p = 0.001], one robust cluster spanning the left OFC, bilateral paracingulate, left caudate and putamen [Z = 4.6; p<0.001], one cluster within the right frontal pole and middle frontal gyrus [Z = 4.4; p<0.001], right caudate and thalamus [Z = 3.9; p = 0.0002] during reward versus control conditions became more positive (see Fig 3A, Table 3, section I). This relationship (between nSLES score and activity in these regions), had no association at lower risk calculator score. Alternatively, activity of bilateral precuneus and superior parietal lobule [Z = 4.4; p<0.001], as well as bilateral central operculum [R: Z = 3.6, p = 2.5e-4; L: Z = 4.0; p = 0.002] showed greater negative associations with nSLES score at increasing risk calculator score (Fig 3B, Table 3, section I). Finally, one region spanning bilateral precuneus and posterior cingulate cortex showed a positive association with nSLES score at low risk calculator score, however, this relationship disappeared as risk calculator score increased [Z = 3.5; p = 0.0005](Table 3, section I). A full set of interaction plots can be found in the supplementary material. Risk calculator score showed a negative relationship with activity in the right supramarginal and angular gyrus [Z = 4.2; p<0.001](Table 3: section I). There were no significant associations between nSLES score and activity during the reward processing task.

**Table 3:** The interaction and main effects of genetic (risk calculator score) and environmental (negative stressful life events score) factors on activity and functional connectivity of reward processing task.

**I. Activity during Reward Processing task**				
**Region**	**x,y,z**	**z**	**p**	**size (# of voxels)**
***Risk calculator score***				
Right supramarginal, angular gyrus	40, -64, 64	4.2	7.8e-8	1692
***Negative stressful life events (nSLE) score***				
No significant results				
***Risk score by nSLE score***				
Left orbitofrontal cortex, bilateral paracingulate, left caudate, putamen, insular cortex	-32, 24, -8	4.6	2.2e-12	3710
Right supramarginal, angular gyrus	46, -62, 60	4.4	1.8e-7	1884
Right frontal pole, middle frontal gyrus	40, 30, 30	4.4	5.0e-5	1156
Right caudate, thalamus	8, 6, 26	3.9	0.00024	971
Left supramarginal, angular gyrus	-46, -62, 58	4.0	0.0014	777
Bilateral precuneus, superior parietal lobule	20, -48, 68	4.4	1.2e-7	1954
Right central operculum, superior temporal, supramarginal cortex	66, 2, 8	3.6	0.00025	966
Bilateral precuneus, posterior cingulate cortex	-6, -56, 24	3.5	0.00053	883
Left central operculum, superior temporal, middle temporal cortex	-62, 6, 2	4.0	0.0016	763
**II. Functional Connectivity during Reward Processing task**				
**Region**	**x,y,z**	**z**	**p**	**size (# of voxels)**
No significant results				

Size is measured as number of voxels (2x2x2mm).

#### Risk calculator score and nSLES score on whole-brain functional connectivity measures during reward processing: Interactions and main effects

No interaction effects or significant associations were found between risk calculator score, nSLES score and functional connectivity with bilateral ventral striatum (seed region) for reward versus control conditions.

## Discussion

This study is the first to identify the interaction and main effects of genetic and environmental risk factors on emotion and reward processing networks within youth at familial risk for future BD. Albeit preliminary, our main findings support our hypothesis that OBP at highest risk of developing BD in the next 5 years, based on predictive risk calculator score, and greater number of recent negative SLEs showed the greatest alterations within the functioning of these emotion and reward processing circuits. We also provided support of the specific contributions of genetic and environmental risk factors on neural functional metrics within OBP.

During emotion processing, higher probability for developing BD in the future was associated with more positive associations between greater exposure to negative SLEs was associated with greater activity in right amygdala, and bilateral fusiform. Lower risk calculator score buffered against positive relationships between nSLES score and activity in these regions. Interestingly, our connectivity results also indicated an importance of amygdala and occipital regions during emotion processing; such that, higher risk calculator score and greater nSLES score was associated with greater functional connectivity of bilateral amygdala and bilateral occipital cortices. Independently, the risk calculator score showed significant positive associations with activity in bilateral lateral occipital cortices, as well as an inverse relationship with functional connectivity between bilateral amygdala and the right lateral occipital cortex during emotion processing. Greater familial risk may be associated with strong effects on amygdala-visual cortical circuitry during emotion processing, which are exacerbated further by exposure to negative SLEs. Although visual processing within the context of emotion processing in BD is not well characterized, there is some support for enhanced recruitment of visual processing regions in BD during the processing of emotional faces [88], as well as evidence of functional coupling of the amygdala and visual cortex in BD and amygdala lesion studies [89, 90]. Behavioral studies have found OBP to have faster response times compared to a HC group, during visual processing tasks [91]. Taken together, OBP might have heightened visual attunement, but it is unclear yet whether it is adaptive or a vulnerability marker.

Individually, greater risk calculator score was positively associated with functional connectivity between bilateral amygdala and bilateral medial orbitofrontal cortex during emotion processing. Previous work in adults with BD is mixed regarding altered functioning of orbital prefrontal network in response to emotion processing [8, 23, 92]. From a developmental perspective, maternal caregiving has been noted to moderate the development of emotion regulation in their offspring via amygdala-mPFC functional connectivity [61]. Typically developing children, in the presence of their caregiver, showed a more adult or “mature-like” amygdala-mPFC functional connectivity pattern compared to children in the absence of their caregiver [61]. The same study noted that in a subset of children showing positive amygdala-mPFC functional connectivity had higher separation anxiety and less secure attachment [61], both of which are thought to reflect future emotional and behavioral problems [93–95]. Our findings may support greater amygdala-OFC functional connectivity as a vulnerability marker for OBP at the greatest risk of developing BD within the next 5 years.

Greater nSLES scores alone were associated with greater positive bilateral amygdala-superior parietal, and amygdala-occipital cortex functional connectivity during emotion processing. These findings might suggest altered visuo-spatial or attentional processing in response to SLEs [96, 97]. There were minimal findings related to the specific contribution of negative SLEs on functional neuroimaging values within the emotion and reward processing networks. This is intuitive as, to date, genetic risk remains the greatest predictor of risk for BD [17, 20].

During reward processing, greater probability for developing BD in the future was associated with more positive relationships between greater exposure to negative SLEs and activity of bilateral superior parietal, paracingulate, striatal, left orbitofrontal and right frontal pole/middle frontal cortices. At lower risk calculator scores, these relationships are not apparent. Independently, greater risk calculator score was negatively associated with activity of the right superior parietal (supramarginal and angular) cortex during reward processing. One interpretation is that as risk for developing BD increases, the ability to buffer against the consequences of exposure to negative SLES is reduced. Previous studies have shown greater VS and left OFC activity in response to reward in BD compared to healthy controls [43], and greater frontal and parietal activity in pediatric BD has been showed in reversal learning [98]. Greater activity of lateral OFC in response to reward in high-risk offspring compared to low-risk offspring has also been observed [47].

Increased risk calculator score was associated with more negative relationships between negative SLEs and activity of bilateral precuneus and superior parietal lobule and bilateral central operculum. As well, one region spanning bilateral precuneus and posterior cingulate cortex showed a positive association with nSLES score at low risk calculator score, however, this relationship disappeared as risk calculator score increased. These findings are more difficult to interpret. Negative associations may reflect a compensatory mechanism in OBP who have no yet developed BD, while these compensatory mechanisms may break down with greater exposure to negative SLEs. Our findings and other previous work support greater engagement of the reward processing network in OBP, and may reflect greater reward or impulsive or sensation-seeking behaviors [99] in those at the greatest risk of developing BD within the next 5 years.

One limitation of our study was our inability to include a control group to the analyses involving future BD risk calculator scores. The risk calculator has been developed for use within OBP populations only [20]. We addressed this limitation by using HCO mean-adjusted values to better interpret the relative neuroimaging activity and connectivity to a normative baseline. Another limitation is that the SLES captures events occurring within the last year. This may vary from year to year, but have more permanent effects within the emotion and reward processing networks. Future studies may aim to investigate the effects of genetic and environmental factors longitudinally to determine which factors are truly important for the development of a disorder. We focused on the impact of familial and environmental risk factors on neural circuitry, but examination of the impact of these risk factors on behavior in OBP can be a focus of future studies. Finally, we must take our findings to be preliminary due to the small sample size and the complexity of these interactions, however, they provide a foundation for future studies to unpack these relationships further.

In summary, while both familial and environmental factors have both been shown to increase risk for BD development, the associations with neural correlates to this point has been unclear. Our findings show distinct associations of familial and environmental risk factors with aberrant functioning of emotion and reward processing networks, but a stronger impact of the interaction between these factors on aberrant functioning in these networks. In line with previous work, genetics may provide the scaffolding to compensate for or buffer against adverse life events. As such, at lower genetic risk, relationships between emotion and reward processing networks were less influenced by recent SLES. However at higher genetic risk, this scaffolding maybe less able to withstand adversity as we saw more aberrant relationships between brain network patterns and exposure to negative SLES. More specifically, higher risk calculator scores were associated with stronger positive relationships between negative SLES score and activity in right amygdala and bilateral fusiform gyri during the emotion processing task, as well as, stronger positive relationships between negative SLES score and activity in the fronto-, striatal, and parietal regions during the reward processing task. Our study identifies potential neural targets to guide the future development of interventions for youth at greatest risk for psychiatric disorders.

## Supporting information

S1 File SupportingMaterials.zip contains a STROBE checklist, the supplementary materials and S1 Fig for this manuscript. (ZIP)

## Abbreviations

ACC: anterior cingulate cortex
BD: bipolar disorder
dlPFC: dorsolateral prefrontal cortex
HCO: healthy control offspring
mPFC: medial prefrontal cortex
nSLES: negative stressful life events schedule
OBP: offspring of bipolar parents
OFC: orbitofrontal cortex
SLEs: stressful life events
VS: ventral striatum
vlPFC: ventrolateral prefrontal cortex
