# Supplementary material for: The impact of familial risk and early life adversity on emotion and reward processing networks in youth at-risk for bipolar disorder
Source: PLoS One. 2019 Dec 12;14(12):e0226135. doi: 10.1371/journal.pone.0226135 (PMC6907842; doi:10.1371/journal.pone.0226135)
Supplement: S1 File — (ZIP) [file pone.0226135.s001.zip › SupportingMaterials_PONE-D-19-03319/Supplementary Materials_PONE-D-19-03319.docx]

**Supplementary Materials**

**Supplementary Methods**

**Additional clinical assessments**

Other collected measures included (1) basic demographic information including age, sex, socioeconomic status, (2) handedness as measured by the Edinburgh Handedness Inventory, (3) acuity as measured by the Shellen Visual Acuity Test, (4) IQ as measured by the Weschler’s Abbreviated Scale of Intelligence (WASI), (5) medications used regularly and within the last 24 hours (of particular importance for psychotropic medications), and (6) drug and alcohol and pregnancy screens.

**Functional imaging tasks**

The dynamic faces task was used to investigate the effects of risk calculator score and exposure to negative stressful life events on implicit emotional processing circuits of the brain. This task has been described previously [1-4]. During the task, participants observe dynamically changing emotional faces while being asked to identify a colour flash that appears within each stimulus. Each face stimulus, taken from the NimStim database, was presented for one second during which time the emotional expression changed in increasing increments of 5% from neutral to one of four full emotions (happy, sad, angry, fearful). Faces were presented in grayscale with oval overlay to remove any background information. The control stimulus was a grayscale oval matched for size and luminance of the face stimuli. A secondary, darker oval was placed on top and dynamically increased in size at the same rate as the face stimuli. Finally, between 200-650ms of each trial, a coloured, semi-transparent coloured oval overlay face or control stimuli. Participants were asked to identify which colour (blue, yellow or orange) was presented within the trial. Task duration was 13 minutes and included: three 12 trial blocks of each emotion type, and twelve 6 trial blocks of the condition stimuli. Each trial was separated by an interstimulus interval of 2-3 seconds.

A number guessing task was used to investigate the effects of risk calculator score and exposure to negative stressful life events on reward circuitry of the brain. This task has been previously described [5-9]. Briefly, participants were asked to guess whether the number about to be presented would be greater or less than 5. With every correct guess, participants were awarded $1 (reward condition), and every incorrect guess, participants lost $0.50 (loss condition). The outcome was fixed; with each participant receiving 15 win trials, 15 loss trials and 18 control trials with total winnings of $10 at the end of the task. The reward condition was signified by a green up arrow being presented during the feedback phase to indicate money awarded. Similarly, during the feedback phase, a red down arrow indicated money lost during the loss condition. Control trials consisted of participants responding when an asterix appeared on the screen. Each trial lasted 7 seconds and consisted of a guessing phase (3 seconds), number presentation (0.5 seconds), feedback phase (0.5 seconds) and a fixed interstimulus interval of 3 seconds.

**Data acquisition**

All images were acquired using a Siemens Magnetom TrimTrio 3T MR system. High-resolution, T1-weighted structural images were acquired with MPRAGE sequence parameters: repetition time (TR) = 2300ms, echo time (TE) = 3.93ms, field of view (FOV) = 256, flip angle (FA) = 9^o^, 192 slices, voxel size = 1.0 x 1.0 x 1.0 mm. Functional images for both tasks were acquired using a gradient-echo, echo-planar sequence: TR = 2000ms, TE = 28ms, FOV = 205, FA= 90^o^, 38 slices, voxel size = 3.2 x 3.2 x 3.1 mm, 386 volumes for the emotion processing task, and 178 volumes for the reward task. Finally, field maps were collected using a gradient-echo sequence: TR = 488ms, TE1 = 4.92ms, TE2=7.38ms, FOV = 256, FA = 60^o^, 32 slices, voxel size = 4.0 x 4.0 x 4.0 mm.

**Additional data analysis and statistics**

For both emotion and reward processing tasks, images were analyzed using FMRIB’s Software Library (FSL:v5.0 [www.fmrib.ox.ac.uk/fsl)](http://www.fmrib.ox.ac.uk/fsl)). Full details on the preprocessing and connectivity pipelines have been previously described [4, 9] in accordance with guidelines set out by the Organization for Human Brain Mapping.

Data processing was carried out using FEAT v6. The preprocessing pipeline included: motion correction using MCFLIRT [10], non-brain tissue removal via BET [11], when possible fieldmap-based echoplanar imaging (EPI) unwarping using PRELUDE+FUGUE [12, 13], spatial smoothing with a FWHM 6mm Gaussian kernel, grand-mean intensity normalization of the entire 4D dataset by a single multiplicative factor, highpass temporal filtering via Gaussian-weighted least-squares straight line fitting (Σ = 50.0s), and local autocorrelation correction using FILM [14]. Coregisteration and normalization of functional images to high-resolution structural and standard MNI space through a three-step process: (1) functional images were registered to structural images using FLIRT (FMRIB’s Linear Image Registration Tool) [10, 15], (2) structural images were registered to the MNI152_T1_2mm standard template using FNIRT (FMRIB’s Non-linear Image Registration Tool [16, 17], (3) both of these transforms were concatenated and applied to the original functional images, resulting in the transform to MNI standard space. Registration quality was checked for each subject, and in very few cases, FNIRT was substituted for FLIRT to improve registration. Next, white matter and cerebral spinal fluid masks, excluding subcortical structures, were generated using fsl_anat, coregistered to the preprocessed functional images, and their time series extracted which were then used as confounding variables at the subject level. Additionally, fsl_motion_outliers were used to detect volumes within the time series that may be corrupted due to excessive motion, and were therefore given a statistical value of zero through the inclusion of a motion outlier matrix at the subject level. Subjects with motion >4mm were excluded. A confounding matrix used at the subject level combined the white matter, cerebral spinal fluid time series and motion outlier matrix.

**References**

1. Perlman SB, Fournier JC, Bebko G, Bertocci MA, Hinze AK, Bonar L, et al. Emotional face processing in pediatric bipolar disorder: evidence for functional impairments in the fusiform gyrus. Journal of the American Academy of Child & Adolescent Psychiatry. 2013;52(12):1314-25. e3.

2. Hafeman D, Bebko G, Bertocci MA, Fournier JC, Chase HW, Bonar L, et al. Amygdala-prefrontal cortical functional connectivity during implicit emotion processing differentiates youth with bipolar spectrum from youth with externalizing disorders. J Affect Disord. 2017;208:94-100. Epub 2016/10/19. doi: 10.1016/j.jad.2016.09.064. PubMed PMID: 27756046; PubMed Central PMCID: PMCPMC5154789.

3. Fournier JC, Keener MT, Almeida J, Kronhaus DM, Phillips ML. Amygdala and whole‐brain activity to emotional faces distinguishes major depressive disorder and bipolar disorder. Bipolar Disorders. 2013;15(7):741-52.

4. Manelis A, Ladouceur CD, Graur S, Monk K, Bonar LK, Hickey MB, et al. Altered amygdala-prefrontal response to facial emotion in offspring of parents with bipolar disorder. Brain. 2015:awv176.

5. Forbes EE, Hariri AR, Martin SL, Silk JS, Moyles DL, Fisher PM, et al. Altered striatal activation predicting real-world positive affect in adolescent major depressive disorder. American Journal of Psychiatry. 2009;166(1):64.

6. Bebko G, Bertocci MA, Fournier JC, Hinze AK, Bonar L, Almeida JR, et al. Parsing dimensional vs diagnostic category–related patterns of reward circuitry function in behaviorally and emotionally dysregulated youth in the longitudinal assessment of manic symptoms study. JAMA Psychiatry. 2014;71(1):71-80.

7. Bertocci MA, Bebko G, Versace A, Fournier JC, Iyengar S, Olino T, et al. Predicting clinical outcome from reward circuitry function and white matter structure in behaviorally and emotionally dysregulated youth. Molecular psychiatry. 2016;21(9):1194-201. Epub 2016/02/24. doi: 10.1038/mp.2016.5. PubMed PMID: 26903272; PubMed Central PMCID: PMCPMC4993633.

8. Bertocci MA, Bebko G, Versace A, Iyengar S, Bonar L, Forbes EE, et al. Reward-related neural activity and structure predict future substance use in dysregulated youth. Psychological medicine. 2017;47(8):1357-69. Epub 2016/12/22. doi: 10.1017/S0033291716003147. PubMed PMID: 27998326.

9. Manelis A, Ladouceur CD, Graur S, Monk K, Bonar LK, Hickey MB, et al. Altered functioning of reward circuitry in youth offspring of parents with bipolar disorder. Psychological Medicine. 2016;46(1):197-208.

10. Jenkinson M, Bannister P, Brady M, Smith S. Improved optimization for the robust and accurate linear registration and motion correction of brain images. Neuroimage. 2002;17(2):825-41.

11. Smith SM. Fast robust automated brain extraction. Human Brain Mapping. 2002;17(3):143-55.

12. Jenkinson M. Fast, automated, N‐dimensional phase‐unwrapping algorithm. Magnetic Resonance in Medicine. 2003;49(1):193-7.

13. Jenkinson M. Improving the registration of B0-disorted EPI images using calculated cost function weights. Neuroimage. 2004;22:e1544-e5.

14. Woolrich MW, Ripley BD, Brady M, Smith SM. Temporal autocorrelation in univariate linear modeling of FMRI data. Neuroimage. 2001;14(6):1370-86.

15. Jenkinson M, Smith S. A global optimisation method for robust affine registration of brain images. Medical Image Analysis. 2001;5(2):143-56.

16. Andersson JL, Jenkinson M, Smith S. Non-linear optimisation. FMRIB technical report TR07JA1. University of Oxford FMRIB Centre: Oxford, UK. 2007.

17. Andersson JL, Jenkinson M, Smith S. Non-linear registration, aka Spatial normalisation FMRIB technical report TR07JA2. FMRIB Analysis Group of the University of Oxford. 2007;2.

**Figures**

**Supplementary Figure 1** – Graphical representations of all significant interaction effects found between risk calculator score, negative Stressful Life Events Schedule (nSLES) score, and (a) function activity during emotion processing (all faces vs shapes contrast), (b) functional connectivity using bilateral amygdala as the seed region during emotion processing (all faces vs shapes contrast), and (c) functional activity during reward processing (reward vs control contrast). Risk calculator score is on the x-axis increasing from left to right, nSLES score is on the y axis increasing from back to front, and the neuroimaging metric on the y-axis increasing from bottom to top. All neuroimaging measures are HCO mean-adjusted values. More details on this can be found in the Methods. Anatomical regions are in order as they appear in Tables 2 and 3, these regions from left to right are: (a) right fusiform cortex, right amygdala, left fusiform cortex, (b) right lateral occipital cortex, left lateral occipital cortex, (c) left orbitofrontal cortex and bilateral paracingulate cortex, right supramarginal and angular gyrus, right frontal pole and middle frontal cortex, right caudate and thalamus, right frontal operculum and orbitofrontal cortex, bilateral precuneus and superior parietal lobule, right central operculum and superior temporal cortex, bilateral precuneus and posterior cingular cortex, and finally, left central operculum and superior temporal cortex.
