# Supplementary figures and images for: The impact of familial risk and early life adversity on emotion and reward processing networks in youth at-risk for bipolar disorder
Source: PLoS One. 2019 Dec 12;14(12):e0226135. doi: 10.1371/journal.pone.0226135 (PMC6907842; doi:10.1371/journal.pone.0226135)

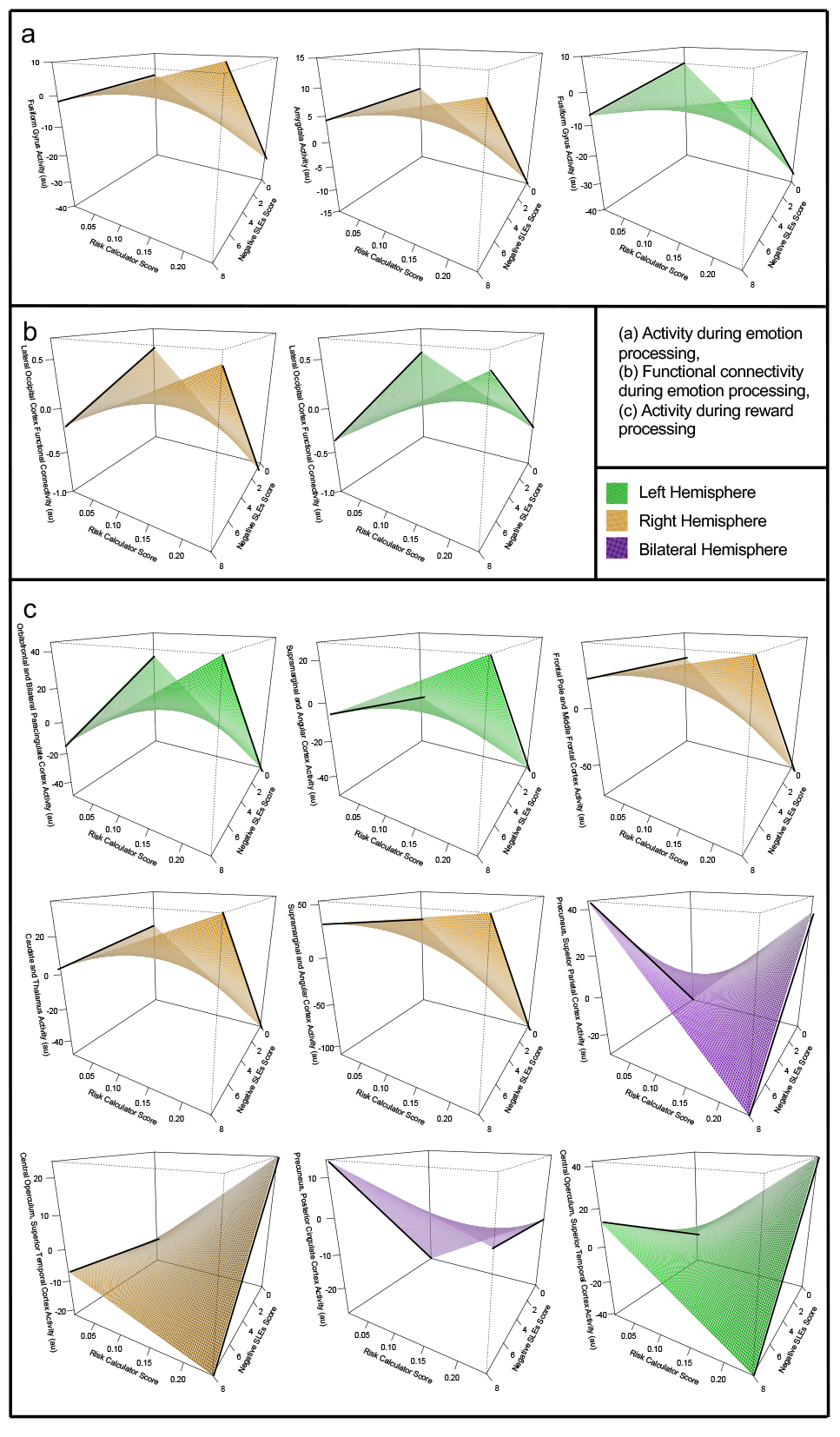

Supplement: S1 File — (ZIP) [file pone.0226135.s001.zip › SupportingMaterials_PONE-D-19-03319/S1Fig_PONE-D-19-03319.tif]
