# Supplementary material for: The impact of familial risk and early life adversity on emotion and reward processing networks in youth at-risk for bipolar disorder
Source: PLoS One. 2019 Dec 12;14(12):e0226135. doi: 10.1371/journal.pone.0226135 (PMC6907842; doi:10.1371/journal.pone.0226135)
Supplement: S1 File — (ZIP) [file pone.0226135.s001.zip › SupportingMaterials_PONE-D-19-03319/STROBE_checklist_PONE-D-19-03319.doc]

**Appendix**

STROBE Statement

|  | | Item No | | Recommendation | | |
| --- | --- | --- | --- | --- | --- | --- |
| **Title and abstract** | | 1 | | (*a*) Indicate the study’s design with a commonly used term in the title or the abstract  **Abstract: Lines 53-57** | | |
|  |  |  |  | (*b*) Provide in the abstract an informative and balanced summary of what was done and what was found  **Abstract: Lines 56-63** | | |
| Introduction | | | | | | |
| Background/rationale | | 2 | | Explain the scientific background and rationale for the investigation being reported  **Introduction: Paragraphs 1-6** | | |
| Objectives | | 3 | | State specific objectives, including any prespecified hypotheses  **Introduction: Paragraph 6** | | |
| Methods | | | | | | |
| Study design | | 4 | | Present key elements of study design early in the paper  **Introduction: Paragraph 6 & Methods: *Risk calculator score, Negative stressful life events schedule (nSLES) score*** | | |
| Setting | | 5 | | Describe the setting, locations, and relevant dates, including periods of recruitment, exposure, follow-up, and data collection  **Methods: *Participants, Clinical assessments***  **Also see Supplementary Materials: *Additional clinical assessments*** | | |
| Participants | | 6 | | (*a*) Give the eligibility criteria, and the sources and methods of selection of participants  **Methods: *Participants, Clinical Assessments*** | | |
|  |  |  |  | (*b*) For matched studies, give matching criteria and the number of controls per case  **NA** | | |
| Variables | | 7 | | Clearly define all outcomes, exposures, predictors, potential confounders, and effect modifiers. Give diagnostic criteria, if applicable  **Methods: *Risk calculator score, Negative stressful life events schedule (nSLES) score, Data analysis, Statistical analyses***  **See also: Supplementary Materials for *Additional data analysis and statistics*** | | |
| Data sources/ measurement | | 8* | | For each variable of interest, give sources of data and details of methods of assessment (measurement). Describe comparability of assessment methods if there is more than one group  **Methods: *Risk calculator score, Negative stressful life events schedule (nSLES) score, Functional tasks***  **See also: Supplementary Materials for *Additional clinical assessments, Functional imaging tasks, Additional data analysis and statistics*** | | |
| Bias | | 9 | | Describe any efforts to address potential sources of bias  **Methods: *Data analysis, Statistical analyses*** | | |
| Study size | | 10 | | Explain how the study size was arrived at  **Methods: Participants *(****no pre-defined study size, all eligible individuals included)* | | |
| Quantitative variables | | 11 | | Explain how quantitative variables were handled in the analyses. If applicable, describe which groupings were chosen and why  **Methods: *Risk calculator score, Negative stressful life events schedule (nSLES) score, Functional tasks***  **See also: Supplementary Materials for *Additional clinical assessments, Functional imaging tasks, Additional data analysis and statistics*** | | |
| Statistical methods | | 12 | | (*a*) Describe all statistical methods, including those used to control for confounding  **Methods: *Statistical analyses*** | | |
|  |  |  |  | (*b*) Describe any methods used to examine subgroups and interactions  **Methods: *Data analysis, Statistical analyses*** | | |
|  |  |  |  | (*c*) Explain how missing data were addressed  **NA** | | |
|  |  |  |  | (*d*) If applicable, describe analytical methods taking account of sampling strategy  **NA** *(this was a convenient sample)* | | |
|  |  |  |  | (*e*) Describe any sensitivity analyses  **NA** | | |
| Results | | | | | |  |
| Participants | 13* | | (a) Report numbers of individuals at each stage of study—eg numbers potentially eligible, examined for eligibility, confirmed eligible, included in the study, completing follow-up, and analysed  **Results: *Demographics and clinical information*** | |  | |
|  |  |  | (b) Give reasons for non-participation at each stage  **Results: *Demographics and clinical information*** | |  | |
|  |  |  | (c) Consider use of a flow diagram  **NA** | |  | |
| Descriptive data | 14* | | (a) Give characteristics of study participants (eg demographic, clinical, social) and information on exposures and potential confounders  **Methods: *Risk calculator score, Negative stressful life events schedule (nSLES) score* & Results: *Demographics and clinical information*** | |  | |
|  |  |  | (b) Indicate number of participants with missing data for each variable of interest  **Methods: *Risk calculator score, Negative stressful life events schedule (nSLES)*** | |  | |
| Outcome data | 15* | | Report numbers of outcome events or summary measures  **Methods: *Risk calculator score, Negative stressful life events schedule (nSLES) score* & Results: *Demographics and clinical information*** | |  | |
| Main results | 16 | | (*a*) Give unadjusted estimates and, if applicable, confounder-adjusted estimates and their precision (eg, 95% confidence interval). Make clear which confounders were adjusted for and why they were included  **Results: *Risk calculator score and nSLES score on whole-brain activity during emotion processing: interactions and main effects, Risk calculator score and nSLES score on whole-brain functional connectivity during emotion processing: interactions and main effects, Risk calculator score and nSLES score on whole-brain activity during reward processing: interactions and main effects, Risk calculator score and nSLES score on whole-brain functional connectivity measures during reward processing: interactions and main effects*** | |  | |
|  |  |  | (*b*) Report category boundaries when continuous variables were categorized  **Methods: *Risk calculator score, Negative stressful life events schedule (nSLES)* & Results: *Risk calculator score and nSLES score on whole-brain activity during emotion processing: interactions and main effects, Risk calculator score and nSLES score on whole-brain functional connectivity during emotion processing: interactions and main effects, Risk calculator score and nSLES score on whole-brain activity during reward processing: interactions and main effects, Risk calculator score and nSLES score on whole-brain functional connectivity measures during reward processing: interactions and main effects*** | |  | |
|  |  |  | (*c*) If relevant, consider translating estimates of relative risk into absolute risk for a meaningful time period  **Methods: *Risk calculator score*** | |  | |
| Other analyses | 17 | | Report other analyses done—eg analyses of subgroups and interactions, and sensitivity analyses  **Results: *Risk calculator score and nSLES score on whole-brain activity during emotion processing: interactions and main effects, Risk calculator score and nSLES score on whole-brain functional connectivity during emotion processing: interactions and main effects, Risk calculator score and nSLES score on whole-brain activity during reward processing: interactions and main effects, Risk calculator score and nSLES score on whole-brain functional connectivity measures during reward processing: interactions and main effects*** | |  | |
| Discussion | | | | | |  |
| Key results | 18 | | Summarise key results with reference to study objectives  **Discussion: Paragraph 1-6** | |  | |
| Limitations | 19 | | Discuss limitations of the study, taking into account sources of potential bias or imprecision. Discuss both direction and magnitude of any potential bias  **Discussion: Paragraph 7** | |  | |
| Interpretation | 20 | | Give a cautious overall interpretation of results considering objectives, limitations, multiplicity of analyses, results from similar studies, and other relevant evidence  **Discussion: Paragraphs 2-6 & 8** | |  | |
| Generalisability | 21 | | Discuss the generalisability (external validity) of the study results  **Discussion: Paragraph 8** | |  | |
| Other information | | | | | |  |
| Funding | 22 | | Give the source of funding and the role of the funders for the present study and, if applicable, for the original study on which the present article is based  **Funding** | |  | |

*Give information separately for cases and controls in case-control studies and, if applicable, for exposed and unexposed groups in cohort and cross-sectional studies.

**Note:** An Explanation and Elaboration article discusses each checklist item and gives methodological background and published examples of transparent reporting. The STROBE checklist is best used in conjunction with this article (freely available on the Web sites of PLoS Medicine at http://www.plosmedicine.org/, Annals of Internal Medicine at http://www.annals.org/, and Epidemiology at http://www.epidem.com/). Information on the STROBE Initiative is available at www.strobe-statement.org.
